# A digital programme to prevent falls and improve well-being in people living with dementia in the community: the KOKU-LITE feasibility randomised controlled trial protocol

**DOI:** 10.1136/bmjopen-2024-091222

**Published:** 2025-08-19

**Authors:** Jaheeda Gangannagaripalli, Emma Vardy, Emma Stanmore

**Affiliations:** 1 The University of Manchester Faculty of Biology Medicine and Health, Manchester, UK; 2 NIHR Applied Research Collaboration Greater Manchester, Manchester, UK; 3 Manchester Academic Health Science Centre, Manchester, UK; 4 Northern Care Alliance NHS Foundation Trust, Salford, UK; 5 School of Health Sciences, The University of Manchester Faculty of Biology Medicine and Health, Manchester, UK

**Keywords:** information technology, dementia, exercise, feasibility studies, health literacy, quality of life

## Abstract

**Introduction:**

Around 885 000 people live with dementia in the UK, of whom around 50% experience a fall each year. ‘Keep On Keep Up’ (KOKU) is a National Health Service-approved gamified, digital health programme designed to maintain function and reduce falls through strength and balance exercises (Fitness and Mobility Exercise/OTAGO Exercise Programme (OTAGO)), and health literacy games. KOKU has been adapted to the needs of people living with dementia in the community (known as KOKU-LITE). This trial aims to test the feasibility and acceptability of trial processes and usability of KOKU-LITE.

**Methods and analysis:**

A two-arm, parallel, non-blinded feasibility randomised controlled trial will be conducted using mixed methods. Participants aged ≥55 years with any type but varying levels of dementia from mild to moderate stages (ratings 1 and 2 on clinical dementia rating (CDR) scale) and meeting the eligibility criteria will be recruited from patient organisations across Greater Manchester, UK. The target sample size is 60 for the trial. Participants randomised into the intervention arm will use the KOKU-LITE programme for 30 min, 3 times a week for 6 weeks plus dementia-specific falls prevention leaflet and participants randomised into the control arm will receive dementia-specific falls prevention leaflet. Outcome measures: primary outcomes: recruitment, retention and adherence rates; secondary outcomes: quality of life, participants’ activities of daily living, physical activity levels, functional ability, lower limb strength, concerns about falling, falls risk, mood and user’s experience of the technology. Post-intervention interviews or focus groups with participants and health and social care professionals will explore the feasibility of trial processes and technology and evaluate the usability and acceptability of the intervention, respectively. Analyses will be descriptive.

Trial status: the first participants were recruited on 20 March 2024. Data collection is currently ongoing.

**Ethics and dissemination:**

This feasibility trial has been reviewed and received favourable ethical approval from Yorkshire & The Humber—Bradford Leeds Research Ethics Committee, Newcastle upon Tyne (REC reference 23/YH/0262). The findings of the study will be disseminated through peer-reviewed scientific journals, at conferences, publication on University of Manchester, Applied Research Collaboration Greater Manchester and KOKU websites.

**Trial registration number:**

NCT06149702.

**STRENGTHS AND LIMITATIONS OF THIS STUDY**

- This is one of the first studies to test the feasibility and acceptability of trial processes and usability of a gamified digital health application for strength and balance training to reduce falls, functional decline and to improve well-being in people living with dementia in the community (known as KOKU-LITE).
- Recruiting and retaining people living with dementia can be very challenging and therefore this feasibility study will explore and evaluate different strategies to recruit participants and estimate the time required for recruitment.
- The barriers and facilitators identified in the recruitment and retention phase of the trial will help us to design a robust definitive trial.
- We have involved people with lived experience of dementia extensively in the development of KOKU-LITE and will continue to do so in feasibility testing of KOKU-LITE to inform a larger study that will test effectiveness.
- This feasibility trial is not powered to determine the effectiveness of the intervention.

## Background

 It is estimated that over 885 000 people with dementia currently live in the UK, with this figure anticipated to increase to 1.6 million by 2040.^1^ The total cost of dementia (including health and social care costs, and opportunity costs of unpaid care) in England in 2015 was £23 billion and is predicted to increase to over £80 billion by 2040.^2^ Due to its increasing prevalence, dementia is recognised as an international priority by WHO.^3^

Falls are a public health concern and a common problem in older people (aged 65 years and over). One in three older adults falls each year,^4^ and those with cognitive impairment are more than twice as likely to fall compared with older adults without cognitive impairment.^5^,^7^ In addition, one in five falls causes serious injuries such as fractures or head injuries.^4^ A recent meta-analysis reported that approximately 44.3% of individuals with Alzheimer’s disease experience at least one fall annually, with an average of 1.30 falls per person per year.^8^ The risk is compounded in older people living with dementia (PLwD) due to additional risk factors.^5 9^ Falls are estimated to cost over £4 billion annually to the UK’s National Health Service (NHS).^10^ Evidence related to interventions that are aimed at reducing falls for PLwD was found to be insufficient and inconclusive.^11^,^13^

There is a growing interest in using digital technologies to support PLwD, and the use is recommended by WHO to promote peoples’ health and well-being.^14^ Using digital technology to enable older PLwD to live independently in their own homes for longer, or to prevent falls, has the potential to improve quality of life and deliver cost savings to health and social care.^15 16^ However, there is a lack of robust evidence to suggest effective digital technologies for PLwD. Eost-Telling *et al*, in their systematic review, found no evidence related to the use of apps for falls prevention in PLwD.^17^ A recent study evaluated the feasibility of a digital falls prevention programme (the StandingTall app) for individuals living with dementia and found the intervention to be both feasible and acceptable in this population. However, this study used a pre-post design without a control group and small sample size.^18^ Evidence also suggests that PLwD are reluctant to use digital technologies due to the following barriers: dementia severity, poor or lack of digital literacy, timing and disease progression, technology anxiety, system failures, digital divide, lack of access to or knowledge of how technology works, cognitive fatigue and usability issues.^19^ Hence, it is crucial that digital interventions are designed in a user-centred manner for technologies to be acceptable.^17 20 21^ Digital technologies have the potential to assist relatives/carers of PLwD in predicting, detecting, monitoring and preventing falls in PLwD.^22 23^

‘Keep on Keep up (KOKU)’ is a tablet/iPad-based digital gamified strength and balance exercise programme for older people at risk of falls and functional decline.^24^ KOKU supports older people to engage with simple, progressive exercises, with the aim of helping people remain functionally active for longer and preventing falls. The strength and balance exercises are based on the evidence-based OTAGO and the Fitness and Mobility Exercise (FaME) programme for community dwelling older adults,^25 26^ which have been shown to reduce falls by around a third. KOKU-LITE is General Data Protection Regulation (GDPR) compliant and has The Organisation for the Review of Care and Health Apps^27^ approval for NHS use in the UK. KOKU-LITE is the dementia-friendly version of KOKU.

## Aim

To test the feasibility of the KOKU-LITE digital programme with PLwD aimed at preventing falls and improving well-being compared with control (a dementia-specific falls prevention leaflet).

## Objectives

1. Explore study recruitment approaches for PLwD, their carers and health and social care professionals (HSCPs) for the feasibility study.
2. Explore the specific training and support needs of PLwD, their carers and HSCPs to enable effective use of KOKU-LITE during the feasibility phase. Particular attention will be paid to the distinct and additional requirements associated with engaging in digital exercises and interactive games on the tablet/iPad, ensuring accessibility and sustained engagement.
3. Explore the usability and acceptability of the KOKU-LITE programme.
4. Describe and determine the suitability of the outcome measures including providing data to permit estimation of effect size to be used in sample size calculations for a definitive trial.

## Methods

### Study design

**Figure 1:**
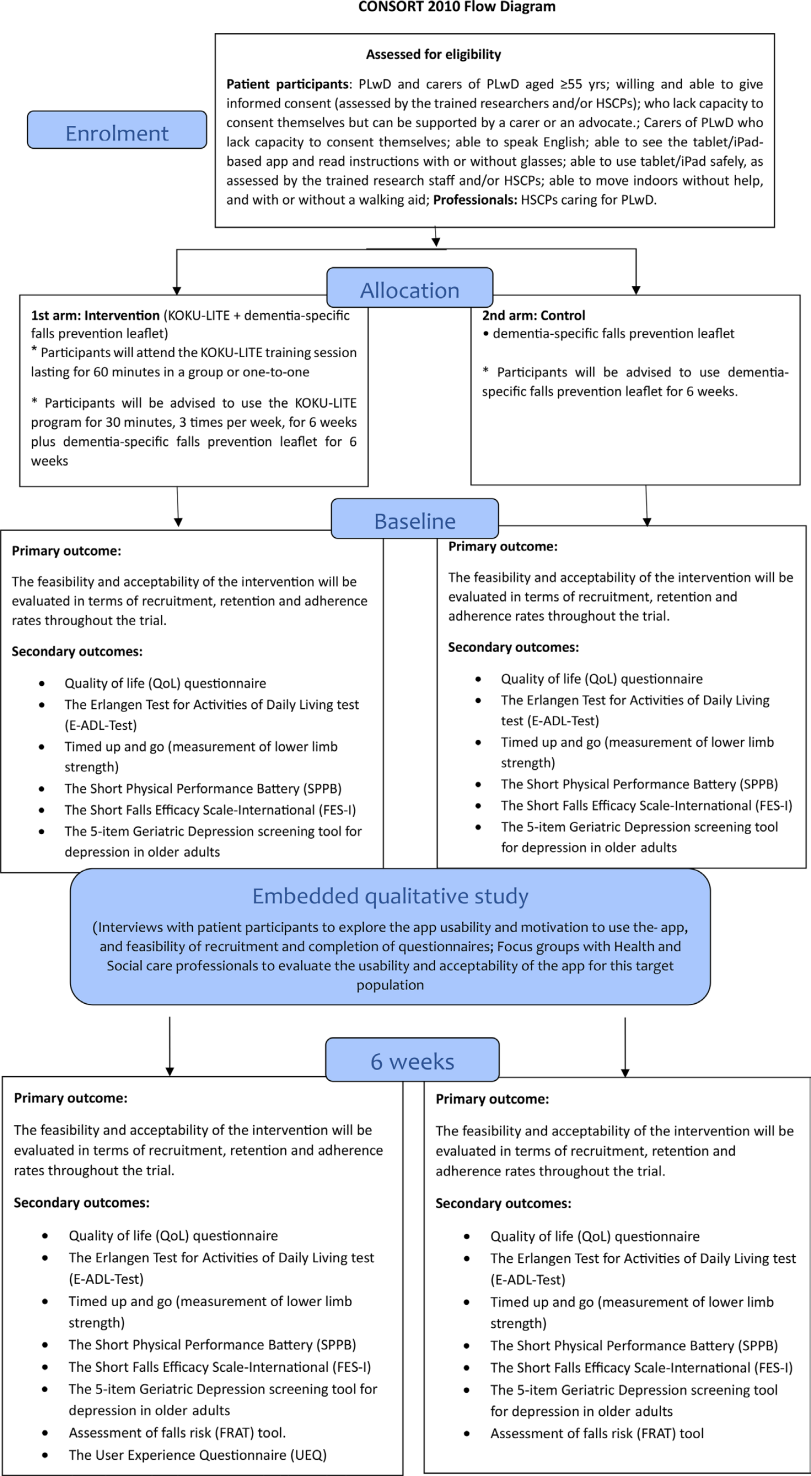
Consolidated Standards of Reporting Trials (CONSORT) flow chart of Keep On Keep Up (KOKU)-LITE feasibility trial. The flow chart demonstrates the participants’ journey from enrolment to follow-up period. HSCP, health and social care professional; PLwD, people living with dementia.

This feasibility randomised controlled trial is a 6-week study comparing the intervention (KOKU-LITE programme+dementia-specific falls prevention leaflet) with control (dementia-specific falls prevention leaflet) in community dwelling PLwD. After assessing participants for eligibility, they will be allocated randomly to either intervention group or control group. Assessments will be performed at baseline and 6 weeks follow-up (figure 1).

A purposive sample of participants who have consented to take part in the study will be invited to participate in postintervention interviews. Exploratory data obtained from the interviews will increase understanding of the responses in the questionnaires about the usability and motivation to use KOKU-LITE, and feasibility of recruitment, data collection approaches and completion of outcome measures.

Patient recruitment has commenced in March 2024 and data collection will be completed by September 2025. The study will be reported in accordance with the Standard Protocol Items: Recommendations for Interventional Trials 2012 Statement for protocols of clinical trials^28^ (online supplemental appendix 1) and TIDieR checklist for the description of the intervention (online supplemental appendix 2). The study process is outlined in figure 1.

### Study participants

As this is a feasibility study, we aim to recruit up to 60 participants (PLwD) (with any type but varying levels of dementia from early diagnosis (mild stages) to moderate stages of dementia (with ratings 1 and 2 on CDR scale) (30 participants in each group) to test the feasibility, usability and acceptability of KOKU-LITE, feasibility of the recruitment processes and suitability of the outcome measures. Up to 15 carers and HSCPs (including general practitioners (GPs), geriatricians, clinical psychologists, psychiatrists, community psychiatric nurses, physiotherapists, occupational therapists, community nurses, occupational therapists and music therapists) (or until saturation) will be recruited to evaluate the usability and acceptability of the app for this target population.

### Participant inclusion/exclusion criteria

#### Inclusion criteria

##### PLwD and carers of PLwD

- PLwD aged ≥55 years.*
- Willing and able to give informed consent (assessed by the trained researchers and/or HSCPs).
- Who lack capacity to consent themselves but can be supported by a carer or an advocate.
- For people with dementia who lack capacity, their carers will be recruited to support them.
- Able to speak English.
- Able to see the tablet/iPad-based app and read instructions with or without glasses.
- Able to use tablet/iPad safely, as assessed by the trained research staff and/or HSCPs.
- Able to move indoors without help, and with or without a walking aid.

*We aim to recruit PLwD aged 55 years and older to ensure that ‘younger-older’ adults with dementia, who may be able to use and benefit from a digital intervention despite cognitive, behavioural and psychological changes, are included.^29^

##### Professionals

- HSCPs caring for PLwD in the community.

### Exclusion criteria

#### People living with dementia

- Participants with:
  - acute/chronic or uncontrolled medical condition (eg, severe congestive cardiac failure, uncontrolled hypertension, acute systemic illness, neurological problems, poorly controlled diabetes);
  - recent fracture or surgery (within 6 months);
  - orthopaedic surgery (such as hip/knee surgery) in the past 6 months or on a waiting list to have the surgery;
  - heart problems such as myocardial infarction or stroke in the past 6 months;
  - conditions requiring a specialist/exercise programme (eg, uncontrolled epilepsy, or uses a wheelchair to mobilise indoors);
  - severe hearing/visual impairment that cannot be corrected with aids;
  - any other medical condition likely to compromise the ability to use/access the app.
- Participants currently in hospital or care home.
- Participants who have limited understanding or ability to speak English.

### Recruitment

#### People living with dementia

*Intervention study*: up to 60 participants (PLwD) (with varying levels of dementia from early diagnosis (mild stages) to moderate stages of dementia) will be recruited. The CDR scale will be used to screen PLwD.^30^ We will work with professional organisations across Greater Manchester, including Dementia United (DU) (DU is Greater Manchester Integrated Care’s programme for dementia), Join Dementia Research (JDR), African Caribbean Care Group, Together Dementia Support, Alzheimer’s Society and Age UK to identify PLwD who are willing and able to participate in the study. The study information, including leaflets and participant information sheet (PIS), will be disseminated on our behalf by DU (established collaboration from previous work), Greater Manchester Mental Health NHS Foundation Trust (GMMH), Age UK, Dementia UK and other patient organisations to their members and the populations that they serve across Greater Manchester.

In addition, we will use snowball sampling (ie, participants will be asked to forward the study leaflet and the PIS to individuals they know who have the same condition) to advertise the study and extend our recruitment to individuals who might not be engaged with third sector organisations. Different formats of the PIS will be prepared (if needed) to accommodate the needs of PLwD. For example, different font sizes and background colours will be used with PIS. Audible versions of the PIS will be provided when required. Participants interested in the study will be advised to contact the research team directly or via their carer (via email) if they are willing to take part in the study. Participants who indicate an interest will then be contacted by the researcher via face-to-face to discuss the study, check eligibility, screen for capacity and answer any questions that participants may have. Assuming participants wish to continue with the study and meet the eligibility criteria, a face-to-face meeting will be arranged either at the participant’s home or a dementia café based on their preference and a time convenient to them. Participants will be sent consent forms (onlinesupplemental appendix 3.1  3.2) in advance of the researcher meeting the them, to ensure they have the opportunity to read the forms and ask any questions. Consent forms will be completed at the participant’s home or at a dementia café based on their convenience.

*Interviews*: up to 15 participants (PLwd) who have expressed an interest to participate in the interview will be contacted by the researcher after the completion of the study via face-to-face or email or telephone based on their preferences to discuss the interview part of the study, and answer any questions that participants may have. All participants will be requested to return the completed consent form (via email or to the researcher for a face-to-face interview) if willing to participate within 1 week of receipt. A follow-up attempt will be made for participants failing to respond within 1 week.

#### Health and social care professionals

*Focus groups*: up to 15 carers, and HSCPs (including GPs, geriatricians, clinical psychologists, psychiatrists, community psychiatric nurses, community nurses, physiotherapists, occupational therapists and music therapists) will be recruited through personal and professional networks including Royal College of General Practitioners, Clinical Research Networks, JDR, Alzheimer’s Society, Age UK and GMMH. HSCPs invited to participate in the study will be provided with a PIS and consent form in advance of their involvement in these events. The study information, including PIS and consent form, will be disseminated on our behalf by the professional organisations. HSCPs who are interested in the study will be advised to contact the researcher, and the researcher will answer any questions the participants may have. All participants will be requested to return the completed consent form if willing to participate within 48 hours if possible. A follow-up attempt will be made for participants who failed to respond within 48 hours; however, extensions can be provided on request to ensure everyone interested has an opportunity to participate.

### Additional recruitment strategy

As an additional strategy to supplement recruitment, the researcher will present information about the study at events organised by patient organisations and local carer groups across Greater Manchester to recruit both PLwD and carers. Participants who indicate an interest to take part in the study will complete a consent-to-contact form. Participants who expressed an interest will be contacted by the researcher via face-to-face or email or telephone as preferred to check eligibility, screen for capacity, discuss the study and answer any questions that participants may have. Assuming participants wish to continue with the study and meet the eligibility criteria, a face-to-face meeting will be arranged either at the participant’s home or a dementia café based on their preference and a time convenient to them. Participants will have 1 week to consider taking part in the study. A follow-up attempt will be made for participants who fail to respond within 1 week.

### Randomisation

After assessing for eligibility, baseline assessments and questionnaires will be undertaken and then participants will be randomly allocated to either receive the intervention (KOKU-LITE programme plus dementia-specific falls prevention leaflet) or control (dementia-specific falls prevention leaflet). Simple randomisation will be done using sealed envelopes^31^ (an online platform to perform randomisation). Randomisation will be performed by a researcher independent of the study. Participants will be informed of the randomisation via telephone, email or post. All participants will have an equal chance of being allocated either to the intervention group (KOKU-LITE programme) or control group (dementia-specific falls prevention leaflet).

### Intervention design

Comparator—a dementia-specific falls prevention leaflet.

The control group will receive a dementia-specific falls prevention leaflet.

#### Study intervention—KOKU-LITE programme

**Figure 2:**
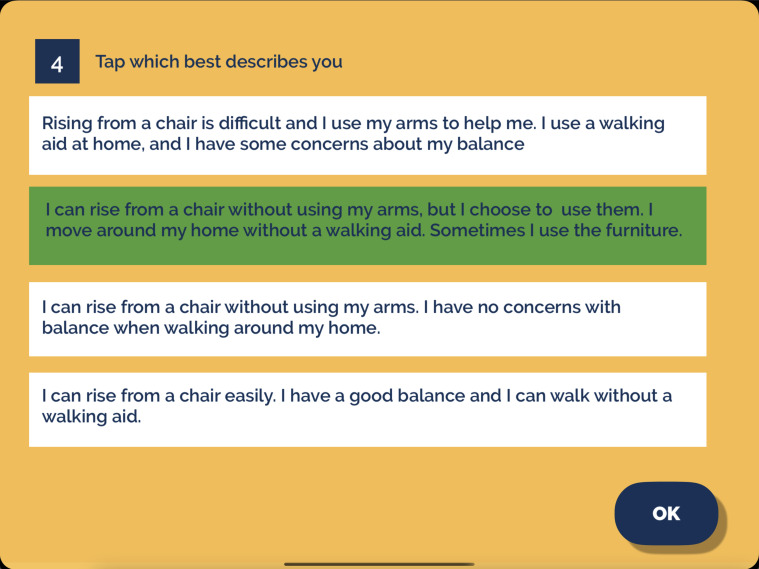
Prescreening questionnaire presented to participants. Participants will be instructed to select an option from the list that best describes them to determine their eligibility for the study.

**Figure 3:**
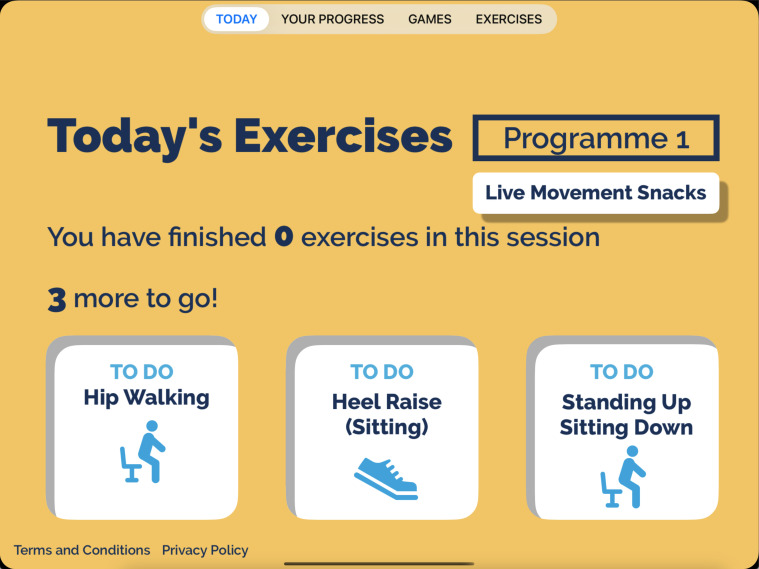
A screen with a set of tailored exercises presented to participants.

The intervention (KOKU-LITE) is a digital gamified strength and balance exercise programme with health literacy games (including home hazard, hydration, nutrition, bone and brain health education). The programme is automatically tailored to the individual needs of each participant. Prior to using KOKU-LITE, participants are advised to complete a prescreening questionnaire (figure 2). Based on their responses, they will be directed to a set of exercises (figure 3) that are appropriately tailored to their functional ability. The exercise regimen begins with simple and low-intensity exercises and progresses gradually to more complex exercises. All exercises are underpinned by the evidence-based principles of FaME-OTAGO strength and balance exercise programmes.

Participants will attend a KOKU-LITE group training session lasting for 60 min. Group sizes may vary between two and four participants, depending on time availability and allocation requirements. A carer may also accompany the participant if they feels their support would be beneficial. An iPad will be provided to participants for the duration of the project and the app instruction booklet will also be provided for reference purposes. For participants who do not wish to attend the group training, ad hoc one-to-one training will be offered.

Participants will be advised to use the modified KOKU-LITE programme for 30 min, 3 times per week, for 6 weeks in their own homes. While we recommend participants to adhere to this dosage, as this is a feasibility study, our primary aim is to assess whether participants are able to follow and adhere to the programme. Evaluating the effectiveness of the intervention will not be the focus of the study at this stage. According to current public health guidelines, it is recommended to engage in moderate to vigorous physical activity for a minimum of 30 min, 3–5 times per week, to promote optimal physical and mental health.^32 33^ Participants will have the flexibility to choose their days and time they use the app, based on their individual needs. Additionally, participants will be encouraged to read and use the dementia falls prevention leaflet alongside the KOKU-LITE app.

During the intervention, the researcher will carry out a weekly visit to participants (either at participants’ home or a dementia café (dementia café is a café that offers a supportive and welcoming space to socialise, learn more about dementia and access helpful resources, information and local services)) based on participants’ preferences to provide support (if required). Additional training on how to use the iPad will be provided in addition to the app training session. During the home visits, researchers will offer personalised support to each participant to ensure adherence to the intervention. Adherence will be monitored through the app’s performance tab (figure 2), which tracks progress and allows for timely adjustments as necessary.

If participants do not adhere to the programme, the following strategies will be implemented:

1. We will investigate the underlying reasons for non-adherence and address these challenges during home visits. In addition, participants will receive weekly phone calls to provide reminders and encourage them to complete the exercises and engage with the games.
2. Based on the identified reasons for non-adherence, we will make appropriate adjustments and arrangements to better support the continued use of the app.

In between the sessions, participants will be advised to contact the research team or dementia support advisors (who organise the cafés) if they experience any difficulties using the app. Contact details of the research team will be provided to the participants.

### Patient and public involvement

KOKU is a tablet/iPad-based digital gamified strength and balance exercise programme, specifically co-designed with older people for older people at risk of falls.^23^ Following Medical Research Council complex intervention guidance,^34^ we have engaged with PLwD and carers of PLwD and co-developed KOKU to suit the needs of PLwD to make it dementia-friendly and more accessible by simplifying the language and other features (online supplemental appendix 4). This version is known as KOKU-LITE. This current project continues the involvement of PLwD and carers of PLwD in feasibility testing of KOKU-LITE to inform a larger study that will test effectiveness.

We have formed a project advisory group from the outset for this study. Project advisory group members have lived experiences, either as a person living with dementia or caring for someone with dementia. Project advisory group members will provide support to the research team throughout the study and with interpretation and dissemination of the findings. Patient and public involvement will be critical and form an integral part of the project to ensure the project discussions are informed by patients’ and carers’ voices. We will explore the support needed for the participants in terms of feasibility and accessibility of the meetings and documentation for the project including protocol, flyers, PIS, consent forms, etc. The group will continuously provide feedback on documents and meet regularly (at least once a month) to support and provide direction to the research.

### Outcome assessment

#### Primary outcomes

The feasibility and acceptability of the intervention will be evaluated in terms of recruitment, retention and adherence rates throughout the trial (table 1). Where possible, reasons underlying these rates will be documented.

**Table 1:** Criteria for progressing to a larger trial

Feasibility	Go—proceed with RCT	Amend—proceed with changes	No go—do not proceed with RCT
Participant recruitment	N≥70 participants consent to the study	N=36–69 participants consent to the study	N≤35 participants consent to the study
Retention rate	≥75% remain in the study (ie, have not withdrawn)*	51%–74% remain in the study (ie, have not withdrawn)	≤50% remain in the study (ie, have not withdrawn)
Data collection	Target: ≥85% of participants from the study have all the study data collected	51%–84% of participants from the study have the study data collected	≤50% of participants from the study have the study data collected
Acceptability			
Engagement and acceptability	≥75% participants used KOKU-LITE intervention	51%–74% participants used KOKU-LITE intervention	≤50% participants used KOKU-LITE intervention

* Retention rate will be ≥75%, considering the patient population.

KOKU, Keep On Keep Up; RCT, randomised controlled trial.

#### Secondary outcomes

The secondary outcomes will be assessed using standardised instruments and assessments (table 2).

**Table 2:** Outcome measures

Outcome	Measure
Quality of life	Measured by The European Quality of Life 5 Dimensions (EQ-5D-5L) scale.^43^ EQ-5D-5L is a five-dimension version of the original EQ-5D scale. It has two sections: the EQ-5D descriptive system and the EQ visual analogue scale (EQ VAS). The descriptive system comprises five dimensions: mobility, self-care, usual activities, pain/discomfort and anxiety/depression. Each dimension has five levels: no problems, slight problems, moderate problems, severe problems and extreme problems. Current recommendations for scoring the EQ-5D-5L index will be followed. Index scores range from −0.59 to 1; 1 is the best possible health state. The EQ VAS records the patient’s self-rated health on a vertical VAS, where 0 indicates ‘worst health you can imagine’ and 100 indicates ‘the best health you can imagine’. Health-related quality of life will be measured at baseline and 6 weeks follow-up.
Activities of daily living	The Erlangen Test for Activities of Daily Living test (E-ADL-Test) is a valid and reliable instrument for assessing the ADL capabilities of people with dementia. It includes four domains: pouring a drink, cutting a piece of bread, opening a little cupboard, washing hands.^44^ The total score of the E-ADL ranges from 0 to 30, with a higher score representing better results. E-ADL-Test will be assessed at baseline and 6 weeks follow-up.
Functional mobility	The Short Physical Performance Battery (SPPB) is a validated and reliable instrument to assess functional mobility in people with cognitive impairment/dementia. The SPPB test includes three different domains: walking, sit-to-stand and balance.^45^ The scores range from 0 (worst performance) to 12 (best performance). Participants will be assessed at baseline and 6 weeks follow-up using SPPB.
Lower limb strength	Timed up and go (measurement of lower limb strength).
Confidence	The Short Falls Efficacy Scale-International (FES-I). The FES-I is a validated and reliable seven-item tool that measures confidence in performing a range of ADL without falling. This scale has recently been modified to maximise its suitability for a range of different languages and cultures.^46^ Scoring: minimum 7 (no concern about falling) to maximum 28 (severe concern about falling). FES-1 scale will be measured at baseline and 6 weeks follow-up.
Depression	The five-item Geriatric Depression Scale (GDS) screening tool for depression in older adults (validated to be as effective as the 15-item GDS for older adults in a wide range of settings.^47^ A score of 0–1 for the five-item GDS suggests that the subject is not depressed, while two or higher indicates possible depression. The five-item GDS screening tool will be measured at baseline and 6 weeks follow-up.
Falls risk	Falls risk will be measured with the use of the assessment of falls risk (FRAT) tool. This validated measure has five items that include: history of any fall in the previous year, four or more prescribed medications, diagnosis of stroke or Parkinson’s disease, reported problems with balance and inability to rise from a chair without using arms. These are all strong indicators for risk of falling.^48^ If a person scores 3 or more, they are at high risk of falling. FRAT tool will be measured at baseline and 6 weeks follow-up.
User experience	The User Experience Questionnaire (UEQ) is a standardised 26-item questionnaire that is used reliably to assess the quality and user experience of the interactive products.^49^ The standard interpretation of the scale means is that values between −0.8 and 0.8 represent a neural evaluation of the corresponding scale, values >0.8 represent a positive evaluation and values <−0.8 represent a negative evaluation. The range of the scales is between −3 (horribly bad) and +3 (extremely good). UEQ will only be measured at 6 weeks postfollow-up in the intervention group only (to those who use KOKU-LITE).

Quality of life will be measured using the European Quality of Life 5 Dimensions scale. In addition to the quality of life, participants’ activities of daily living (ADL) physical activity levels, functional ability, lower limb strength, fear of falling, falls risk, mood and user’s experience of the technology (KOKU-LITE app in this study) will be measured. Vision will be assessed using a self-reported question: “At the present time, would you say that your eyesight, using both eyes (with glasses or contact lenses if you wear them), is” (response options: excellent, good, fair, poor, very poor, registered blind).

#### Participant characteristics

Medical history (including fractures), medication comorbidities and demographic data (age, gender, ethnicity, socioeconomic status, etc) will also be recorded.

### Sample size

The total sample size for this study is 75 (60 PLwD for the feasibility study and 15 HSCPs for the interviews).

The intended sample size for the feasibility study will be 30 PLwD in each group, in which simple randomisation will be used with a 1:1 allocation. An attrition rate of 10%–20% is reasonable. A formal sample size calculation is not required as this study focuses on the practicalities of conducting the study. This number is pragmatic to assess the feasibility of the KOKU-LITE programme with the outcome measurements. It is anticipated that this sample size will provide sufficient information to inform a future definitive trial, assuming that the KOKU-LITE programme proves feasible and acceptable for PLwD.^35^

Up to 15 carers and HSCPs (including GPs, geriatricians, clinical psychologists, psychiatrists, community psychiatric nurses, community nurses, physiotherapists, occupational therapists and music therapists) will be recruited to evaluate the usability and acceptability of the app for this target population.

Feasibility of the KOKU-LITE intervention will be assessed based on the feasibility criteria outlined in table 1.^35^,^38^

### Blinding

Blinding of participants and researchers and outcome assessors will not be possible in this study due to the nature of the intervention and due to the lack of resources.

### Data collection

#### Quantitative research

Demographic data and standardised assessments/questionnaires will be completed at baseline and 6 weeks from both the intervention and control groups in participants’ homes by a member of the research team. Additional questionnaires about usability and motivation to use the app will also be completed at 6 weeks. Due to resource constraints, the same researchers will be responsible for delivering the intervention and collecting assessments/outcomes data. Follow-up outcomes data will be collected even if participants discontinue the use of the KOKU-LITE app.

#### Qualitative research

After 6 weeks of follow-up period, up to 15 participants who have expressed an interest from both the arms will be approached to take part in a semi-structured interview to:

1. talk about how the study was carried out and whether improvements could be made to the recruitment strategy, PIS, etc;
2. discuss their views about the usability and acceptability of the KOKU programme. The interview will last approximately 60 min.

Carers and HSCPs (including GPs, geriatricians, clinical psychologists, admiral nurses, occupational therapists, etc) will be invited to attend up to three focus groups, lasting up to 60 min. The aim of the focus group is to gather their views about how usable and acceptable the app might be for PLwD, and any changes they would suggest making it suitable for this population.

Semi-structured interviews and focus groups will be conducted either face-to-face or through online platforms (such as MS Teams or Zoom), according to each interviewee’s preference (and in compliance with any restrictions in place due to COVID-19). Interviews or focus groups will last up to 60 min. Interviews or focus groups will be recorded (audio) with participant’s permission. If an online platform is used, participants will be asked to turn off the video after the researcher and participant introductions. The topic guides used to conduct the interviews will be informed by the literature and our previous work. The topic guide (for PLwD and carers) will be piloted with the Project Advisory Group.

### Analysis

#### Quantitative research

Analysis will be descriptive. Baseline characteristics and postintervention data will be summarised, as appropriate, using means (with SD) or median (IQR). For dichotomous or categorical data, frequency will be reported by number or percentage of responses within each category. Calculations will be conducted using SPSS V.29.

As this is a feasibility study, formal hypothesis testing will not be conducted for secondary outcomes, which include changes in health-related quality of life, ADL, physical activity, functional ability and other fall-related measures. Instead, intervention effects will be summarised using point estimates and their corresponding SDs. These estimates will be calculated as the unadjusted mean differences between baseline and each postintervention timepoint. The resulting summary statistics will inform sample size calculations for a future definitive trial, if progression criteria are met. Basic statistical comparisons of pre-intervention and post-intervention outcomes will be performed to describe trends, applying an intention-to-treat approach to ensure that participants are analysed according to their original group allocation.

#### Qualitative research

The interviews and focus groups will be transcribed verbatim by the research team and then imported into NVivo V.14 for analysis. Thematic analysis will be undertaken following the stages outlined by Clarke *et al*.^39^ Line-by-line coding will be conducted, leading to a thematic analysis. Following data familiarisation, initial codes will be generated and peer-reviewed by a member of the research team to identify common themes. The analysis will be carried out using NVivo software V.14. The thematic analysis will broadly explore the acceptability and feasibility of the study design and the intervention, from the perspectives of PLwD as well as HSCPs. The first few interviews (of each group) will be subject to independent coding to achieve consistency.

### Data monitoring and quality assurance

This study will be subject to the audit and monitoring regime of the University of Manchester (UoM). Governmental monitoring agencies may also access study data for monitoring and auditing purposes. A data monitoring committee will not be used.

If there are any adverse events or evidence that would cause the researcher to electively stop the research project prematurely, the researchers will make a decision to stop the trial; however, this decision will be based on serious adverse events (SAEs). However, no adverse events were reported as a direct result of being involved in the previous KOKU trials. We will stop the trial if the SAE rate in the intervention group is >15% higher than the control group.

### Ethics and dissemination

#### Ethical considerations

The study will be conducted in full conformance with principles of the ‘Declaration of Helsinki’, Good Clinical Practice and within the laws and regulations of the UK.

The main ethical considerations associated with this project relate to the involvement of PLwD and some of their carers (if of older age) and as such, they are potentially vulnerable. Therefore, the researcher will work closely with patient organisation representatives to ensure that the processes that we put in place are deemed appropriate, suitable and acceptable and incur minimal participant burden to this vulnerable population.

At least 1 week before the study initiation, participants will receive PIS describing the study, their voluntary participation and how their personal data and rights will be protected. Informed consent will be obtained before their participation. A distress protocol will be created and adhered to during the study.

#### Informed consent

Capacity to consent is of utmost importance in individuals with dementia; therefore, we will endeavour to support PLwD in consenting for their participation in research. We will adhere to principles of the Mental Capacity Act^40^ and the UK Network of Dementia Voices (DEEP) gold standards for dementia research.^41^ We will abide by the rules and principles of ethical research and informed consent outlined in the DEEP-Ethics gold standards document recognising for ethical research and informed consent, which have been informed by PLwD. Written consent will be obtained from all participants. If a person with dementia is unable to provide a signed consent form, then verbal consent will be obtained. PIS will be developed in easy-read and accessible formats (large font, lay language, short sentences combined with images) if needed. The information sheets will be reviewed by members of the project advisory group (who are not participants in the study).

### Protocol amendments

Any modifications to the protocol will be immediately notified to the sponsor and necessary amendments will be obtained from the ethics committee.

### Data handling and management

The rights of the participants will be protected in terms of privacy, confidentiality, pseudo-anonymity and minimum required information in accordance with GDPR. Access to the participants’ information will be restricted to members of the research team. The data will be pseudonymised (participants’ names will not be used and any identifying information will be removed and replaced with a unique ID number).

Electronic data, including audio consent recordings, will be held on UoM’s network drives on a password-protected computer owned by the UoM accessed from the research team’s own homes (due to hybrid working). Paper-based data will be stored in a digital locked filing cabinet in the research team’s office at the UoM. Audio consent forms will be transcribed by the research team. Assessments, questionnaires, signed consent forms, transcripts of audio consent and permission to contact forms will be digitised and stored on the UoM’s secure database on network drives in a password-protected computer as soon as practicable.

Participants’ contact details will be deleted at the end of 2 years. All other data will be kept for 5 years in line with the University regulations. Electronic data will be permanently deleted from web-based electronic databases/drives after this period. Paper-based data will be shredded according to University policies. Participant details will not be passed onto any third parties.

### Dissemination

The results of the study will be disseminated through traditional routes including peer-reviewed publications and at conferences, through patient organisations newsletters and websites and through social media. To increase the public understanding of the study, the study outputs will be presented at public events organised by dementia-specific patient and third sector organisations.

Impact at international level will be achieved through UoM Applied Research Collaboration and KOKU website, which might include descriptions of the study and app prototype, and future work.

## Discussion

To our knowledge, this is one of the first trials that explores the feasibility and acceptability of trial processes and usability of a gamified digital health application for strength and balance training to reduce falls, functional decline and to improve well-being in PLwD in the community. The KOKU-LITE programme has been modified by incorporating feedback from patient and public involvement and engagement representatives to suit their needs (online supplemental appendix 1). The exercises in the programme will start slowly and progress with the user, thereby aiming to build confidence and reduce concerns about falling. Early testing as part of the iterative development process of KOKU-LITE showed that participants valued having the remote facility to exercise and enjoy the health literacy games without the need for the internet. They also found it to be accessible and user-friendly. Participants liked the animated character that demonstrated the exercises for them and reported this motivated them to perform the exercises. Our study will further explore and examine how the KOKU-LITE programme can be delivered effectively in the community dwelling PLwD.

We will recruit participants from across the spectrum (including individuals with different types of dementia and different stages of the disease, ethnicity, socio-economic status) to evaluate benefits of the intervention on quality of life and well-being, and to cater for a range of physical abilities of PLwD. This will also provide us with an opportunity to gain an understanding of the feasibility of the participants with communication difficulties filling out the questionnaires. We will also investigate the feasibility of different outcome measures used in this study, which will add to the body of work on outcome measures for this population. This will also aid in the development of new measures if the existing ones are found to be impractical.

The intervention participants will receive weekly home visits. To support the sustainability of the intervention, we will adhere to the recommendations outlined by Mortan *et al*^42^: foster understanding and support from trusted friends, family and health professionals, as their encouragement can be key for long-term sustainability. Be sensitive to differences in abilities, ages and stages and aim to empower members rather than avoid challenges for them. Stay in constant contact with potential referrers and keep them involved. Outside of a study setting, digital literacy and access to tablets/iPads would need to be considered for larger scale-up of the programme.

We have actively engaged in participant recruitment through community organisations fostering strong relationships with these groups. Dementia advisors, who oversee the coordination of dementia cafés, have expressed their support by helping to remind participants and their carers about the continued use of the intervention during these sessions. These interactions also provide valuable opportunities for collaboration with both participants and community organisations to explore and develop a range of strategies.

Blinding of participants and researchers will not be possible in this study due to the nature of the intervention; however, randomisation will be performed by a researcher independent of the study.

The barriers and facilitators identified in the recruitment and retention phase of the trial will help us to design a robust definitive trial.

## Supplementary material

10.1136/bmjopen-2024-091222 online supplemental file 1

10.1136/bmjopen-2024-091222 online supplemental file 2

10.1136/bmjopen-2024-091222 online supplemental file 3

10.1136/bmjopen-2024-091222 online supplemental file 4

10.1136/bmjopen-2024-091222 online supplemental file 5
