# Supplementary material for: A digital programme to prevent falls and improve well-being in people living with dementia in the community: the KOKU-LITE feasibility randomised controlled trial protocol
Source: BMJ Open. 2025 Aug 19;15(8):e091222. doi: 10.1136/bmjopen-2024-091222 (PMC12366566; doi:10.1136/bmjopen-2024-091222)
Supplement: online supplemental file 4 [file bmjopen-15-8-s004.docx]

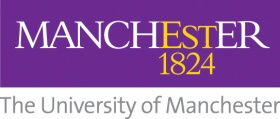

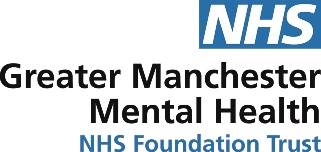


**A feasibility randomised controlled trial of a digital program to prevent falls and improve well-being in people living with Dementia in the community.**

**Keep-on-Keep-up (KOKU)-LITE**

**Participant Consent Form (written)**

If you are happy to participate please complete and sign the consent form below.

|  | **Activities** | Initials |
| --- | --- | --- |
| 1 | I confirm that I have read and understood the information sheet **Version 2.4; 14.01.2025** for the above study. I have had the opportunity to consider the information and ask questions and have had these questions answered to my satisfaction. |  |
| 2 | I understand that my participation in the study is voluntary and that I am free to withdraw at any time without giving a reason and without detriment to myself. I understand that it will not be possible to remove my data from the project once it has been anonymised and forms part of the data set. I agree to take part on this basis. |  |
| 3 | I understand that data collected about me will be stored securely and in pseudonymised form. |  |
| 4 | I agree that any data collected may be used in future studies and published in anonymous form in academic books, reports journals or conference presentations |  |
| 5 | I understand that a fully anonymised dataset will be deposited in an open data repository at the end of the project. |  |
| 6 | I understand that data collected during the study may be looked at by individuals from The University of Manchester, from the NHS or regulatory authorities, where it is relevant to my taking part in this research. I give permission for these individuals to have access to my data. |  |
| 7 | I understand that details about me including name, address, bank details will be passed to the University's Finance team for the sole purpose of processing payments |  |
| 8 | I understand that there may be instances where during the course of the research information is revealed which means the researchers will be obliged to break confidentiality and this has been explained in more detail in the information sheet. |  |
| 9 | I agree to take part in the study |  |

**The following activities are optional, you may participate in the research without agreeing to the following:**

| 10 | I agree that the researchers may contact me in future about other research projects. |  |
| --- | --- | --- |
| 11 | I agree that the researchers may retain my contact details in order to provide me with a summary of the findings for this study. |  |

**Data Protection**

**The personal information we collect and use to conduct this research will be processed in accordance with data protection law as explained in the Participant Information Sheet and the** [**Privacy Notice for Research Participants**](http://documents.manchester.ac.uk/display.aspx?DocID=37095)**.**

______________________ ________________________

Name of Participant Signature Date

______________________ ________________________

Name of Witness Signature Date

________________ ______________

Name of the person taking

declaration Signature Date

1 copy for the participant, 1 copy for the research team (original)

form will be retained by the research team and you will be given a copy of this form.
