# Supplementary material for: A digital programme to prevent falls and improve well-being in people living with dementia in the community: the KOKU-LITE feasibility randomised controlled trial protocol
Source: BMJ Open. 2025 Aug 19;15(8):e091222. doi: 10.1136/bmjopen-2024-091222 (PMC12366566; doi:10.1136/bmjopen-2024-091222)
Supplement: Supplementary file 6 [file bmjopen-2024-091222.reviewer_comments.pdf]

PEER REVIEW HISTORY

BMJ Open publishes all reviews undertaken for accepted manuscripts. Reviewers are asked to complete a checklist review form and are provided with free text boxes to elaborate on their assessment. These free text comments are reproduced below.

ARTICLE DETAILS

Title (Provisional)

A digital programme to prevent falls and improve well-being in people living with dementia in the community: the KOKU-LITE feasibility Randomised Controlled Trial protocol

Authors

Gangannagaripalli, Jaheeda; Vardy, Emma; Stanmore, Emma

VERSION 1 - REVIEW

**Reviewer**	**1**
**Name**	**Taylor, Morag**
**Affiliation**	**Neuroscience Research Australia, Falls, Balance and Injury Research Centre**
**Date**	**26-Nov-2024**
**COI**	**None**

Title: A digital program to prevent falls and improve well-being in people living with dementia in the community: the KOKU-LITE feasibility Randomised Controlled Trial protocol

Manuscript ID: bmjopen-2024-091222

Overall

This is a protocol paper for a feasibility RCT of a digital exercise program adapted for people with dementia. Sixty older people living with dementia have been recruited, of which up to 15 will be interviewed post-intervention. Carers and health professionals will also be recruited for focus groups. This is a carefully thought-out study with fairly robust methods. I have a few comments that I feel will improve the protocol.

1. Paragraph 2 of background: this paragraph is based on 65 years and older, but your inclusion criteria is 55 years and older – suggest some comment is needed in this paragraph to support recruiting younger participants.

2. P6, line 38: "6 weeks post follow up" – this could be written more clearly as from this statement it is not clear if it is 6-weeks post baseline or 6-weeks post intervention.
3. Study participants: 'service users' is used but is not defined. Suggest it should be defined as it not clear why a PLwD is a service user.
4. For inclusion criterion "Carers of PLwD who lack capacity to consent themselves." I think this can be interpreted two ways – 1) the person with dementia lacks capacity or 2) the carer lacks capacity, so it could be written "For people with dementia who lack capacity, their carers will be recruited". Or something similar as I initially interpreted it that you were recruiting carers who lacked capacity – which I don't think you mean?
5. I feel that the recruitment section and randomisation section should appear earlier in the methods. Café's are mentioned in the intervention section, but the reader has no understanding of what these are. Simple randomisation appears in the sample size section, but then the description of this appears later in the randomisation section.
6. The intervention could be better described in the main document: is it automatically tailored and progressive, give some examples of the types of exercises, how they are presented etc. A table providing this detail could be included with the main manuscript, but the current TIDIER statement does not describe the intervention in enough detail – e.g. it does not indicate how tailoring will be achieved, what type of exercises are delivered using the app etc.
7. Monitoring of adherence could be described in the manuscript – particularly since it is a primary outcome measure. What happens if a person does not adhere is there any follow-up mechanism? Or will this be addressed in the weekly home visits?
8. It would be good to provide a justification for your approach to the dose of the exercise program.
9. How will feasibility be determined using recruitment, retention and adherence – e.g. what level will be acceptable for retention and adherence for the trial to be deemed feasible? What rate of recruitment will be seen as meeting the feasibility criteria? As these are the primary outcomes they should be defined.
10. I would suggest in the secondary outcomes section that the reference to Table 1 should come earlier – preferably at the beginning.
11. P10 quantitative analysis – will any contrasts be made between baseline and post-intervention data? If so, how and will you use intention-to-treat? When referring to 'outcome data' do you mean post-intervention data?
12. P12 "Thematic analysis will be undertaken using following the stages outlined in Braun and Clarke (38)" – did you mean to include the stages here?
13. Data handling and management – should data retention be mentioned here?
14. P14 discussion: there has been a digital exercise program previously tested for feasibility for people with dementia living in the community – which is mentioned in the discussion of one of your references: Eost-Telling: systematic review of systematic reviews; the manuscript was published after the last review – hence why it is mentioned in the discussion. So, one could argue that, although there is still merit in conducting the research, that this is not the first feasibility trial of a digital exercise for people with dementia.
15. The intervention participants will receive weekly home visits – some comment in the discussion on the sustainability of this model would be useful, or plan for supporting longer term use. Particularly since the program (at 1.5h/week for 6 weeks) will not meet the suggested dose for fall prevention, appreciate that this is not dementia specific.
16. SPIRIT statement – I don't feel that criteria 11a, 11c and 12 are sufficiently met within the manuscript. Some statements are missing from the manuscript and could be included e.g. 11d, 18b, 20c and 25

Minor comments:

P10, line 13 – should failed by failing?

P12, lines 6 and 11: please review [check]

Suggest PAG could be spelled out for ease of readability.

Reviewer **2**

Name **Schoene, Daniel**

Affiliation **Robert Bosch Hospital, Department of Clinical Gerontology**

Date **24-Mar-2025**

COI **None**

The manuscript entitled ‚A digital program to prevent falls and improve well-being in people living with dementia in the community the KOKU-LITE feasibility Randomised Controlled Trial protocol’ describes an interesting and needed study that will help to get further insight into the feasibility, usability and acceptability of an app-based intervention in PLwD. Knowledge gained will inform a future clinical trial. There are only few comments that may be addressed to avoid misunderstandings and enhance reporting.

Abstract:

Include sample size/number of people targeted

Include additional information on how often/long App is supposed to be used (30 minutes, 3 times per week)

Specify primary outcomes

Introduction:

“Falls are estimated to cost over £4 billion annually (10).” Please specify country/region.

Objectives: “2) Explore the training and support required for PLwD, their carers and HSCPs for the use of KOKU-LITE during the feasibility study.” More information could be provided on how this objective will be operationalised.

Methods

“Intervention: KOKU-LITE program + a Dementia specific-falls prevention leaflet

Control: a Dementia specific-falls prevention leaflet” Why are these written in bold under the header ‘Design’? It doesn’t seem to fit this section.

“Patient recruitment has commenced in March 2024 and data collection will be completed by September 2024.” I reckon this is a typo and the authors mean September 2025, also in

accordance with trial registry entry. Otherwise, journal policy doesn't allow publishing protocols after completion of the recruitment phase.

Study participants: please provide detail on dementia staging within the study ("[…] from early diagnosis to moderate stages of Dementia") as well as Dementia types included.

Study intervention: from experience, one group training session at the beginning in this target group may not be sufficient for all participants. Are there plans how to handle this issue? What is the group size?

Images of the intervention could help readers to get a better understanding of the App, its handling and the exercises.

Primary Outcomes: Please provide more information on measures of primary outcomes. Text and Table 1 don't include information. Apologies if I overlooked anything.

Will adherence be measured via he tablets? If yes, what information will be obtained? If no, how will adherence be measured?

Page 8 line 14 change to "trial"

Recruitment: "All participants will be requested to return the completed Consent Form if willing to participate within 48 hours." This is not a lot of time and may preclude interested people from participating.

Randomisation: "If participants are allocated to the intervention group, they will use KOKU-LITE for 30 minutes 3 times a week for 6 weeks plus Dementia specific falls prevention leaflet. If participants are allocated to the control group, they will receive Dementia specific falls prevention leaflet (and not use the KOKU-LITE intervention)." Redundant as stated before

Blinding: "Blinding of participants and researchers will not be possible in this study due to the nature of the intervention and due to the lack of resources." Please specify intervention providers/instructors (or similar) and outcome assessors.

Data collection: Why is the following under the header 'Quantitative data'? It appears to deal with qualitative data:

"* After 6 weeks, a maximum of 15 participants who has expressed an interest will be approached to take part in a semi structured interview to:

a) talk about how the study was carried out and whether improvements could made to the recruitment strategy, PIS etc. and

b) discuss their views about the usability and acceptability of the app. The interview will last approximately 60 minutes.

*Carers, and Health and social care professionals (including General Practitioners (GPs), geriatricians, clinical psychologists, admiral nurses, occupational therapists etc) will be

invited to attend up to 3 focus groups, lasting up to 60 minutes. The aim of the focus group is to gather their views about how usable and acceptable the app might be for PLwD, and any changes they would suggest making it suitable for this"

Analysis: "Calculations will be conducted using SPSS version 24 [check]." And "The interviews and focus groups will be transcribed verbatim by the research team and then imported into NVivo V. [check] for analysis" Please check and remove the "[check]"

Patient and public involvement: as the KOKU and KOKU-Lite Apps were developed in close collaboration with older people and PLwD, this could be placed in this paragraph

Data handling and management should include brief information on deletion/destruction of data.

Discussion

P13 L10-11: there by to thereby

P13 L14: found it "to" be […]

**Reviewer**	**3**
**Name**	**Said, Catherine**
**Affiliation**	**The University of Melbourne, Physiotherapy**
**Date**	**02-Apr-2025**
**COI**	**None**

Thank you for providing me with the opportunity to review this well written protocol for an important issue, falls prevention in people living with dementia.

While overall it is clearly written, there are a number of points that require clarification.

Please clarify the dates of commencement and completion. The date of commencement was March 2024; and it is planned to be completed by September 2024. Given it is now March 2025, please confirm that the study is still underway and not completed. Please provide a trial status update (eg actual date recruitment commenced, number of people recruited at time of submission) and revise anticipated completion date.

Abstract: You have listed a number of outcomes in the methods section. It may be useful to differentiate between primary and secondary outcomes.

Objectives:

How are you evaluating objectives 1) and 2)? None of the outcomes appear to directly address these objectives.

Re objective 4, note that there are some concerns about relying on data from pilot trials to calculate sample size (see https://www.nccih.nih.gov/grants/pilot-studies-common-uses-and-misuses).

Methods:

As it currently reads, there are three headings all at the same level with no text under each heading (Study design, Intervention and Control). I am not sure that the Intervention and Control headings are required here; this information can be incorporated into the description of the study design.

Inclusion criteria: Clarify which inclusion criteria are 'and' and which are 'or'. For example, 'willing and able to give informed consent OR lack capacity to consent themselves but can be supported by a carer'. Clarify whether you are including people with any type of dementia? Are you collecting data on type of dementia? Do you require any confirmation from a medical practitioner that the participant has been diagnosed with dementia, or is it via self-report? Are you doing any cognitive tests to get an indication of dementia severity?

Secondary outcomes: Some of the assessments included are not really outcome measures (eg past medical history). Consider differentiating these under a separate heading (eg. Participant characteristics)

Interviews: Will all these participants be recruited from the intervention arm?

Baseline Assessments: Where will these be conducted, by whom?

Blinding: While it is not possible to blind participants and researchers involved in the intervention, it is possible to blind those collecting outcome data. However, I assume there is insufficient funding to allow this. Please make this clearer.

Data collection. Clarify whether carers/ health professionals are required to attend up to 3 focus groups or only 1 focus group (with up to 3 focus groups in total being held). Will the carers/ health professionals have had exposure/ used the app before the focus group

Data monitoring: How will data on AE/ SAE be collected? Will you be collecting this data from those in the control group as well? Are you collecting data on falls during the intervention period?

Trial registration: Please provide the date of registration.

SPIRIT Checklist: Please include a statement on the role of study funders.

Appendix 1:

Daily exercises put pressure on people. The response in the Changes to be implemented column is not clear. When you refer to '…participants suggested LESS is MORE', is this referring to the amount of content, or exercise? Is this a change you intend to make (as suggested by the column heading), or have you decided not to included this?

There are a number of minor typographical errors :

Pg 8: line 43 '….. the PIS to individuals who they know of the same condition'.

Pg 10 line 25 '…. A maximum of 15 participants who has…'

Pg 11 line 11 [check] '…will be undertaken using following the stages..'

Pg 13 line 10 '… progress with the user there by building confidence ….'

Formatting

There are some minor formatting issues:

• Please revise levels of headings.

• page 11 line 25 and line 35 the paragraphs start with an asterisk. Is this necessary?

VERSION 1 - AUTHOR RESPONSE

Reviewer #1		
1	Paragraph 2 of background: this paragraph is based on 65 years and older, but your inclusion criteria is 55 years and older – suggest some comment is needed in this paragraph to support recruiting younger participants.	Many thanks for highlighting this. We have now addressed your query under the eligibility criteria section rather than the background section and justified this. Please let us know if you would prefer this to be moved back to the background section—we are happy to adjust accordingly (please see page 6).
2	P6, line 38: "6 weeks post follow up" – this could be written more clearly as from this statement it is not clear if it is 6-weeks post baseline or 6-weeks post intervention.	Thanks for spotting this. We have now clarified the wording to make it clearer that this is post-baseline and updated it as 'Assessments will be performed at baseline and 6 weeks follow up' (please see page 5).
3	Study participants: 'service users' is used but is not defined. Suggest it should be defined as it not clear why a PLwD is a service user.	Apologies for the confusion. We have removed the term 'service users' completely and replaced it with people living with dementia (PLwD) throughout.
4	For inclusion criterion "Carers of PLwD who lack capacity to consent themselves." I think this can be interpreted two ways – 1) the person with dementia lacks capacity or 2) the carer lacks capacity, so it could be written "For people with dementia who lack capacity, their carers will be recruited". Or something similar as I initially interpreted it that you were recruiting carers who lacked capacity – which I don't think you mean?	Many thanks for this suggestion – we have now rephrased the sentence as suggested: 'For people with dementia who lack capacity, their carers will be recruited'(please see page 6).
5	I feel that the recruitment section and randomisation section should appear earlier in the methods. Café's are mentioned in the intervention section, but the reader has no understanding of what these are. Simple randomisation appears in the sample size section, but then the description of this appears later in the randomisation section.	We have moved the recruitment and randomisation section earlier in the methods section – thank you for your helpful suggestion.<br>We have now included a clear definition of a Café (please see page 10).
6	The intervention could be better described in the main document: is it automatically tailored and progressive, give some examples of the types of exercises, how they are presented etc. A table providing this detail could be included with the main manuscript, but the current TIDIER statement does not describe the intervention in enough detail – e.g. it does not indicate how tailoring will be achieved, what type of exercises are delivered using the app etc.	We have now included both text and figures to show the app's content and have provided a detailed description of the intervention (please see page 9).
7	Monitoring of adherence could be described in the manuscript – particularly since it is a primary outcome measure. What happens if a	Thank you for the suggestion – we have now included text about how adherence is recorded by the digital

	person does not adhere is there any follow-up mechanism? Or will this be addressed in the weekly home visits?	intervention to address the points you raised (please see page 10).
8	It would be good to provide a justification for your approach to the dose of the exercise program.	We have included justification and supporting references for the dosage of our exercise programme in the section Study Intervention – KOKU-LITE program (please see pages 9&10). .
9	How will feasibility be determined using recruitment, retention and adherence – e.g. what level will be acceptable for retention and adherence for the trial to be deemed feasible? What rate of recruitment will be seen as meeting the feasibility criteria? As these are the primary outcomes they should be defined.	Thank you for your query. We have provided the required justification criteria to enable us to progress with a larger trial, see table 2 (please see pages 12 and 28).
10	I would suggest in the secondary outcomes section that the reference to Table 1 should come earlier – preferably at the beginning.	Thank you for the suggestion. We have moved the reference to table 1 to the beginning of the sentence (please see page 11).
11	P10 quantitative analysis – will any contrasts be made between baseline and post-intervention data? If so, how and will you use intention-to-treat? When referring to 'outcome data' do you mean post-intervention data?	We will conduct basic statistical analysis on both pre and post-intervention data describe trends using intention-to-treat analysis. Apologies for the confusion – yes, we are referring to the post-intervention data (please see page 15).
12	P12 "Thematic analysis will be undertaken using following the stages outlined in Braun and Clarke (38)" – did you mean to include the stages here?	Apologies for the oversight, we have now included the stages outlined by Braun and Clarke (please see page 15).
13	Data handling and management – should data retention be mentioned here?	We have added text on data retention in this section (please see page 17).
14	P14 discussion: there has been a digital exercise program previously tested for feasibility for people with dementia living in the community – which is mentioned in the discussion of one of your references: Eost-Telling: systematic review of systematic reviews; the manuscript was published after the last review – hence why it is mentioned in the discussion. So, one could argue that, although there is still merit in conducting the research, that this is not the first feasibility trial of a digital exercise for people with dementia.	Thank you for raising this – we have revised the sentence to specify 'gamified digital health app' using a tablet/iPad instead of a 'digital program' (please see page 18).
15	The intervention participants will receive weekly home visits – some comment in the discussion on the sustainability of this model would be useful, or plan for supporting longer term use. Particularly since the program (at 1.5h/week for 6 weeks) will not meet the	We have added text in the discussion section to support the weekly home visits model and sustainability of the programme within the study as well as longer term sustainability (please see page 18).

	suggested dose for fall prevention, appreciate that this is not dementia specific.	
16	16. SPIRIT statement – I don't feel that criteria 11a, 11c and 12 are sufficiently met within the manuscript. Some statements are missing from the manuscript and could be included e.g. 11d, 18b, 20c and 25.	We have included the relevant information in the appropriate sections and updated the SPIRIT checklist accordingly.
17	P10, line 13 – should failed by failing?	Apologies for this typo – we have corrected this now as ' A follow-up attempt will be made for participants who fail to respond within one week' (please see page 8).
18	P12, lines 6 and 11: please review [check]	We have updated the versions for SPSS and NVivo software – thank you for spotting this (please see page 15).
19	Suggest PAG could be spelled out for ease of readability.	We have spelled PAG throughout – thank you for your suggestion.
**Reviewer #2**		
1	Abstract:<br>Include sample size/number of people targeted<br>Include additional information on how often/long App is supposed to be used (30 minutes, 3 times per week)<br>Specify primary outcomes	Many thanks for the suggestion. We have included the sample size and additional information to the abstract, and have also specified the primary outcomes.
2	Introduction:<br>"Falls are estimated to cost over £4 billion annually (10)." Please specify country/region.	We have now specified the country – thank you for the suggestion (please see page 4 paragraph 2).
3	Objectives: "2) Explore the training and support required for PLwD, their carers and HSCPs for the use of KOKU-LITE during the feasibility study." More information could be provided on how this objective will be operationalised.	We have now provided more information on this objective (please see page 5).
4	Methods:<br>"Intervention: KOKU-LITE program + a Dementia specific-falls prevention leaflet<br>Control: a Dementia specific-falls prevention leaflet" Why are these written in bold under the header 'Design'? It doesn't seem to fit this section.	We have incorporated this into the study design description as below:<br>" This feasibility randomised controlled trial is a 6-week study comparing the intervention (KOKU-LITE program + Dementia specific-falls prevention leaflet) to control (Dementia specific-falls prevention leaflet) to control in community dwelling PLwD" (please see page 5).
5	Patient recruitment has commenced in March 2024 and data collection will be completed by September 2024." I reckon this is a typo and the authors mean September 2025, also in accordance with trial registry entry. Otherwise, journal policy doesn't allow publishing protocols after completion of the recruitment phase.	Apologies for the oversight – we have now updated the data collection period (please see page 6).

6	Study participants: please provide detail on dementia staging within the study ("[…] from early diagnosis to moderate stages of Dementia") as well as Dementia types included.	Many thanks for raising this query. We have clarified the dementia staging and the types of dementia (please see page 6).
7	Study intervention: from experience, one group training session at the beginning in this target group may not be sufficient for all participants. Are there plans how to handle this issue? What is the group size?	We agree with the reviewer, and thank you for raising this. We have included supporting text to clarify this point. Group sizes may vary between 2 and 4 participants, depending on the time availability and allocation considerations (please see page 9).
8	Primary Outcomes: Please provide more information on measures of primary outcomes. Text and Table 1 don't include information. Apologies if I overlooked anything.	Many thanks for your query. We have added additional text to support this outcome, and have also included more information on feasibility outcomes in the sample size section (please see page 11).
9	Will adherence be measured via he tablets? If yes, what information will be obtained? If no, how will adherence be measured?	Yes. There is a performance tab in the app as shown in figure 3, where users' performance will be monitored (please see page 10).
10	Page 8 line 14 change to "trial"	Thanks for spotting the error – we have corrected this now.
11	Recruitment: "All participants will be requested to return the completed Consent Form if willing to participate within 48 hours." This is not a lot of time and may preclude interested people from participating.	We have included information on the further extension to ensure fair participation (please see page 8).
12	Randomisation: "If participants are allocated to the intervention group, they will use KOKU-LITE for 30 minutes 3 times a week for 6 weeks plus Dementia specific falls prevention leaflet. If participants are allocated to the control group, they will receive Dementia specific falls prevention leaflet (and not use the KOKU-LITE intervention)." Redundant as stated before	We have deleted this now.
13	Blinding: "Blinding of participants and researchers will not be possible in this study due to the nature of the intervention and due to the lack of resources." Please specify intervention providers/instructors (or similar) and outcome assessors.	We have clarified this sentence which now reads as follows: Due to limited resources, the same researchers will be responsible for delivering the intervention and collecting assessments/outcomes data (please see page 14).
14	Data collection: Why is the following under the header 'Quantitative data'? It appears to deal with qualitative data:<br>"* After 6 weeks, a maximum of 15 participants who has expressed an interest will be approached to take part in a semi structured interview to:	Thanks for this query – we have now moved this to qualitative data section (please see page 14).

	a) talk about how the study was carried out and whether improvements could made to the recruitment strategy, PIS etc. and<br>b) discuss their views about the usability and acceptability of the app. The interview will last approximately 60 minutes.<br>*Carers, and Health and social care professionals (including General Practitioners (GPs), geriatricians, clinical psychologists, admiral nurses, occupational therapists etc) will be<br>invited to attend up to 3 focus groups, lasting up to 60 minutes. The aim of the focus group is to gather their views about how usable and acceptable the app might be for PLwD, and any<br>changes they would suggest making it suitable for this"	
15	Analysis: "Calculations will be conducted using SPSS version 24 [check]." And "The interviews and focus groups will be transcribed verbatim by the research team and then imported into NVivo V. [check] for analysis" Please check and remove the "[check]"	Thanks for spotting this. We have corrected and updated the software versions (please see page 15).
16	Patient and public involvement: as the KOKU and KOKU-Lite Apps were developed in close collaboration with older people and PLwD, this could be placed in this paragraph	Many thanks for this suggestion. We have moved the relevant statements to the Patient and public involvement section (please see page 10).
17	Data handling and management should include brief information on deletion/destruction of data.	Many thanks for this query. We have now added text regarding the deletion/destruction of the data (please see page 17).
18	P13 L10-11: there by to thereby	Apologies for this typo – we have corrected this now (please see page 18).
19	P13 L14: found it "to" be […]	Apologies for the typo – we have added "to" the sentence (please see page 18).
	**Reviewer #3**	
1	Please clarify the dates of commencement and completion. The date of commencement was March 2024; and it is planned to be completed by September 2024. Given it is now March 2025, please confirm that the study is still underway and not completed. Please provide a trial status update (eg actual date recruitment commenced, number of people recruited at time of submission) and revise anticipated completion date.	Apologies for the confusion. We have updated the status of the trial in the study design section (please see page 6).
2	Abstract: You have listed a number of outcomes in the methods section. It may be useful to differentiate between primary and secondary outcomes.	Thank you for the suggestion. We have differentiated between the primary and secondary outcomes.

3	Objectives:<br>How are you evaluating objectives 1) and 2)? None of the outcomes appear to directly address these objectives.<br>Re objective 4, note that there are some concerns about relying on data from pilot trials to calculate sample size (see https://www.nccih.nih.gov/grants/pilot-studies-common-uses-and-misuses [nccih.nih.gov]).	We are collecting data on recruitment and retention rates and conducting qualitative interviews to explore recruitment approaches/strategies and training needs.<br>Thank you for the advice and for the resource. We will work with our statistician to determine the appropriate sample size (please see page 5).
4	As it currently reads, there are three headings all at the same level with no text under each heading (Study design, Intervention and Control). I am not sure that the Intervention and Control headings are required here; this information can be incorporated into the description of the study design.	Thanks for the suggestion – we have incorporated the information about the intervention and control groups into the description of the study design (please see page 5).
5	Inclusion criteria: Clarify which inclusion criteria are 'and' and which are 'or'. For example, 'willing and able to give informed consent OR lack capacity to consent themselves but can be supported by a carer'. Clarify whether you are including people with any type of dementia? Are you collecting data on type of dementia? Do you require any confirmation from a medical practitioner that the participant has been diagnosed with dementia, or is it via self-report? Are you doing any cognitive tests to get an indication of dementia severity?	Apologies for any confusion. We are recruiting individuals living with dementia who have the capacity to provide consent and participate in the study, as well as individuals who lack capacity but can still be involved in the study. While assessments will be conducted with people with dementia, the information about them will be completed by their carers. We have clarified the information about types of dementia as follows:<br>We are including participants with any time of dementia and collect information on various types of dementia. The Clinical Dementia Rating (CDR) will be used to assess dementia severity, and assessments will be carried out by healthcare professionals within the team, including geriatricians, pharmacists and nurses." (please see page 6)
6	Secondary outcomes: Some of the assessments included are not really outcome measures (eg past medical history). Consider differentiating these under a separate heading (eg. Participant characteristics)	Many thanks for this suggestion. We have now separated outcome measurements and participant characteristics under separate headings for clarity (please see page 11).
7	Interviews: Will all these participants be recruited from the intervention arm?	No, we will be recruiting participants from both arms of the study, and we have clarified this in the manuscript (please see page 5).
8	Baseline Assessments: Where will these be conducted, by whom?	Baseline assessments will be conducted by one of the researchers at participants' homes. We have clarified this in the manuscript (please see page 14).

9	Blinding: While it is not possible to blind participants and researchers involved in the intervention, it is possible to blind those collecting outcome data. However, I assume there is insufficient funding to allow this. Please make this clearer.	Many thanks for this query. We have clarified this and included supporting text under the quantitative data collection section (please see page 14).
10	Data collection. Clarify whether carers/ health professionals are required to attend up to 3 focus groups or only 1 focus group (with up to 3 focus groups in total being held). Will the carers/ health professionals have had exposure/ used the app before the focus group	We will only conduct one focus group with carers/healthcare professionals- with a total of up to 3 focus groups planned.<br>Yes, we will demonstrate the app to the carers and healthcare professionals before the focus group session.
11	Data monitoring: How will data on AE/ SAE be collected? Will you be collecting this data from those in the control group as well? Are you collecting data on falls during the intervention period	Adverse events (serious or non-serious) will not be collected for this study. However, we have included information regarding serious adverse events, and we will make every effort to stop the trial in event of a serious incident. Yes, we are collecting data on falls (please see page 16).
12	Trial registration: Please provide the date of registration.	We have provided the trial registration date now (please see page 19).
13	SPIRIT Checklist: Please include a statement on the role of study funders.	We have included text on role of the study funders (please see page 24).
14	Appendix 1:<br>Daily exercises put pressure on people. The response in the Changes to be implemented column is not clear. When you refer to '…participants suggested LESS is MORE', is this referring to the amount of content, or exercise? Is this a change you intend to make (as suggested by the column heading), or have you decided not to included this?	Apologies for the confusion. This refers to the content and not the exercises. We are providing verbal guidance to participants when introducing KOKU-LITE and during training, instead of including more text in the app itself.
15	There are a number of minor typographical errors :<br>Pg 8: line 43 '….. the PIS to individuals who they know of the same condition'.<br>Pg 10 line 25 '…. A maximum of 15 participants who has…'<br>Pg 11 line 11 [check] '…will be undertaken using following the stages..'<br>Pg 13 line 10 '… progress with the user there by building confidence ….'	Apologies and thank you for spotting the typos: we have now corrected all of them (please see pages 7, 14, 15, and 18).
16	Formatting<br>There are some minor formatting issues:<br>• Please revise levels of headings.<br>• page 11 line 25 and line 35 the paragraphs start with an asterisk. Is this necessary?	Many thanks for identifying these formatting issues. We have made the necessary amendments.

VERSION 2 - REVIEW

**Reviewer**	**1**
**Name**	**Taylor, Morag**
**Affiliation**	**Neuroscience Research Australia, Falls, Balance and Injury Research Centre**
**Date**	**16-Jun-2025**
**COI**	

Title: A digital program to prevent falls and improve well-being in people living with dementia in the community: the KOKU-LITE feasibility Randomised Controlled Trial protocol

Manuscript ID: bmjopen-2024-091222.R1

Thanks to the authors for revising the manuscript, which I feel is improved.

I have a number of remaining suggestions for consideration:

1. The primary outcome of the trial is not included in the CONSORT diagram (seems to focus on secondary outcomes).

2. Page 4, line 38 – should this be preventing?

3. Page 6, line 32 – suggest saying dementia, instead of 'the condition'

4. P10 line 44 – reference to table 2 should probably be included here.

5. Please include name of trial registry in the abstract.

6. Ideally the abstract would include inclusion criteria, blinding, date of first recruitment and trial status.

7. In the discussion (p15), I would suggest that you use the wording you have used on page 3 – 'one of the first trials that explores the feasibility of ….', as the previous feasibility trial of a digitally delivered exercise program also used iPads/tablets and provided progressive exercise to people with dementia. The gamified components of KOKU lite seem to be eg. Locating home hazards vs actual gamification of the exercises. However, this is not clear without seeing the app.

8. The previous feasibility trial in people with dementia, nor any of the other manuscripts reporting on the efficacy of the StandingTall app, have been referenced in the protocol.

9. Some of the references are quite old e.g. those related to fall prevalence and risk for people with dementia – these could be updated with more recent literature.

**Reviewer**	**2**
**Name**	**Schoene, Daniel**
**Affiliation**	**Robert Bosch Hospital, Department of Clinical Gerontology**
**Date**	**01-Jun-2025**
**COI**	

Thanks for updating the manuscript according to the comments by reviewers. Good luck with the research!

VERSION 2 - AUTHOR RESPONSE

Query no.	Query text	Response
	**Reviewer #1**	
1	The primary outcome of the trial is not included in the CONSORT diagram (seems to focus on secondary outcomes).	Thank you for highlighting this, and apologies for the oversight. The primary outcome has now been added to the CONSORT diagram.
2	Page 4, line 38 – should this be preventing?	Thanks for spotting this. We have now corrected the wording by changing from 'prevention' to 'preventing'
3	Page 6, line 32 – suggest saying dementia, instead of 'the condition'	Thanks for the suggestion. We have now replaced 'condition' with 'dementia'
4	P10 line 44 – reference to table 2 should probably be included here.	We have included a reference to Table 2.
5	Please include name of trial registry in the abstract.	We have added the name of the trail registry in the abstract.
6	Ideally the abstract would include inclusion criteria, blinding, date of first recruitment and trial status.	Thank you for the suggestion. We have now included additional details on the inclusion criteria, blinding, date of first recruitment and trial status to the abstract.
7	In the discussion (p15), I would suggest that you use the wording you have used on page 3 – 'one of the first trials that explores the feasibility of ….', as the previous feasibility trial of a digitally delivered exercise program also used iPads/tablets and provided progressive exercise to people with dementia. The gamified components of KOKU lite seem to be eg. Locating home hazards vs actual gamification of the exercises. However, this is not clear without seeing the app.	Thanks for the suggestion. We have now added the same wording from page 3 to the discussion section as recommended.
8	The previous feasibility trial in people with dementia, nor any of the other manuscripts reporting on the efficacy of the StandingTall app, have been referenced in the protocol.	Many thanks for highlighting this. We have now added text about the feasibility trial of the StandingTall app in the background section (page 4 paragraph 3).
9	Some of the references are quite old e.g. those related to fall prevalence and risk for people with dementia – these could be updated with more recent literature.	We have now updated the references to include recent literature.
